# Impact of pro-domain stability of matrix metalloproteinase-8 on the outcome of sepsis

**DOI:** 10.1186/cc9698

**Published:** 2011-03-11

**Authors:** J McLaughlin, J Rella, A Bakan, L Kong, L Zhu, D Frederick, S Yende, R Ferrell, I Bahar, S Shapiro, D Angus, A Kaynar

**Affiliations:** 1 University of Pittsburgh, PA, USA; 2 University of Vienna, Austria; 3 University of Tulane, New Orleans, LA, USA

## Introduction

Animal studies suggest matrix metalloproteinase-8 (MMP8) (neutrophil collagenase) impairs neutrophil (PMN) recruitment in inflammation; in humans, MMP8 has been associated with inflammation. We hypothesized that septic patients with single nucleotide polymorphisms (SNPs) in the MMP8 promoter region will have a survival advantage, and this advantage is due to differences in MMP8 enzymatic activity and not MMP8 levels.

## Methods

We examined data from patients with CAP-associated sepsis (GenIMS), analyzed three functional SNPs (rs3765620, rs1940475, rs11225395) in 1,567 Caucasians and tested associations with 60-day and 90-day mortality and severe sepsis incidence. We simulated functional MMP8 SNPs using anisotropic network modeling. Modeling suggested pro-domain structural stability affecting zymogen activation. Based upon the predictions, we then studied zymogen activation using bioluminescent resonance energy transfer (BRET). We generated recombinant pro-MMP8 with a pro-domain tag of luciferase and carboxy terminus tag of green fluorescent protein. BRET signal was generated when luciferase-cleaved substrate produced a photon transferring energy to the GFP acceptor. GFP in turn emitted a green light signal when the donor/acceptor pairs were spatially close. Upon MMP activation, pro-domain is cleaved causing a loss in BRET signal.

## Results

The rs1940475 genotype causing an amino acid mutation in the pro-domain was significantly associated with 90-day mortality (AA: 8.5%, AG: 11.1%, GG: 14.7%, *P *= 0.007). Cumulative incidence showed that the A allele was associated with better 90-day survival. Computer simulation of the mutation suggests a delayed activation. BRET assay confirmed that pro-domain mutation of MMP8 (K87E) rendered it less amenable to activation.

## Conclusions

Our results suggest altering the structural stability of the inhibitory MMP8 pro-domain impacts enzyme activation. Therapeutics targeting pro-domain could be used to modulate MMP function and control downstream inflammatory processes in sepsis.

